# Sox2 function as a negative regulator to control HAMP expression

**DOI:** 10.1186/s40659-015-0013-z

**Published:** 2015-05-06

**Authors:** Bin Song, Qi Bian, Cheng-Hao Shao, An-An Liu, Wei Jing, Rui Liu, Yi-Jie Zhang, Ying-Qi Zhou, Gang Li, Gang Jin

**Affiliations:** Department of General Surgery, Changhai Hospital, Second Military Medical University, Shanghai, 200433 China; Department of Nephrology, Changhai Hospital, Second Military Medical University, Shanghai, 200433 China

**Keywords:** Sox2, Gene expression, Hepatocyte, HAMP, Iron metabolism

## Abstract

**Background:**

Hepcidin, encoding by HAMP gene, is the pivotal regulator of iron metabolism, controlling the systemic absorption and transportation of irons from intracellular stores. Abnormal levels of HAMP expression alter plasma iron parameters and lead to iron metabolism disorders. Therefore, it is an important goal to understand the mechanisms controlling HAMP gene expression.

**Results:**

Overexpression of Sox2 decrease basal expression of HAMP or induced by IL-6 or BMP-2, whereas, knockdown of Sox2 can increase HAMP expression, furthermore, two potential Sox2-binding sites were identified within the human HAMP promoter. Indeed, luciferase experiments demonstrated that deletion of any Sox2-binding site impaired the negative regulation of Sox2 on HAMP promoter transcriptional activity in basal conditions. ChIP experiments showed that Sox2 could directly bind to these sites. Finally, we verified the role of Sox2 to negatively regulate HAMP expression in human primary hepatocytes.

**Conclusion:**

We found that Sox2 as a novel factor to bind with HAMP promoter to negatively regulate HAMP expression, which may be further implicated as a therapeutic option for the amelioration of HAMP-overexpression-related diseases, including iron deficiency anemia.

**Electronic supplementary material:**

The online version of this article (doi:10.1186/s40659-015-0013-z) contains supplementary material, which is available to authorized users.

## Background

Hepcidin, encoding by HAMP gene, is the key regulator of iron homeostasis, and is a small, defensin-like peptide produced by the liver [1,2]. HAMP expression is tightly regulated by many signals including iron content, erythropoietic activity, and inflammation [3-5]. The expression of the HAMP is increased in patients with anemia of chronic disease. Anemia of chronic disease (ACD), also known as anemia of inflammation, is the most prevalent type of anemia in hospitalized patients worldwide [6,7]. The pathogenesis of ACD is characterized by iron-restricted erythropoiesis, whereas iron is retained in the macrophages leading to the disorder of total body iron [8,9]. It has now become clear that inflammatory cytokines released during acute infection or chronic disease can alter systemic iron metabolism by inducing excess synthesis of hepcidin [4,7,8,10]. Treatment of anemia, when necessary, has included administration of iron, packed red cell transfusion, or erythropoiesis-stimulating agents. However, concerns over adverse effects of these therapies, including iron overload, increased risk of infection, recurrence of cancer, and cardiovascular complications, have driven the need for alternative treatments [7,11,12]. Due to the central role of HAMP as described above, inhibition of its biological activity or its expression level may be promising new approaches for the treatment of anemia associated disease.

Sox2 is a member of the Sox family of transcription factors. This protein family shares highly conserved DNA binding domains known as HMG (High-mobility group) box domains containing approximately 80 amino acids [13]. Sox2 is essential for maintaining self-renewal, or pluripotency, of undifferentiated embryonic stem cells [14-16]. We analyzed the HAMP promoter region and putative Sox2 binding sites were identified, suggesting that Sox2 maybe play roles to regulate HAMP expression. In current study, we found that Sox2 functions as the negative regulator to bind with the HAMP promoter to control the HAMP expression. Overexpression of Sox2 in Huh7 cells or HepG2 cells can significantly decrease HAMP expression; in contrast, knockdown Sox2 with siRNA can further increase HAMP expression. We also identified two putative Sox2 binding sites in HAMP promoter and generated the HAMP promoter driven luciferase reporter construct. With this reporter construct, we found that both the Sox2 binding sites are required for Sox2 to exert its negative regulation on HAMP expression; we demonstrated that Sox2 can directly bind with the putative binding sites with ChIP assay. Finally, we also found that Sox2 can downregulate HAMP expression in primary hepatocytes.

In conclusion, our study presents important evidence for the regulation of HAMP expression by Sox2, and this finding can supply a potential therapeutic option to treat anemia of chronic disease.

## Results and discussion

### Overexpression of Sox2 decrease HAMP expression in hepatoma-derived cells

We searched HAMP expression with BioGPS database (http://biogps.org/) and the analysis suggest that HAMP is highly and specifically expressed in liver tissue (Additional file 1: Figure S1). This result is validated in many cell lines from different tissues with by RT-qPCR assay. As shown in Additional file 1: Figure S2, HAMP is highly expressed in cell lines derived from hepatocytes.

Anemia of chronic disease is a condition caused by increased HAMP production, and is a result of inflammation [7,8]. The central role of HAMP in the maintenance of iron homeostasis suggests that targeting of this peptide may result in therapeutic treatments of iron-overload disorders, so we analyzed the promoter region, aiming to find the transcription factor to control HAMP expression. TFsearch program (http://www.cbrc.jp) and TESS Tool (http://www.cbil.upenn.edu/cgi-bin/tess/tess) analysis suggest that putative Sox2 binding sites were identified in this region, thus, we hypothesize that Sox2 can control the expression of HAMP.

**Figure 1:**
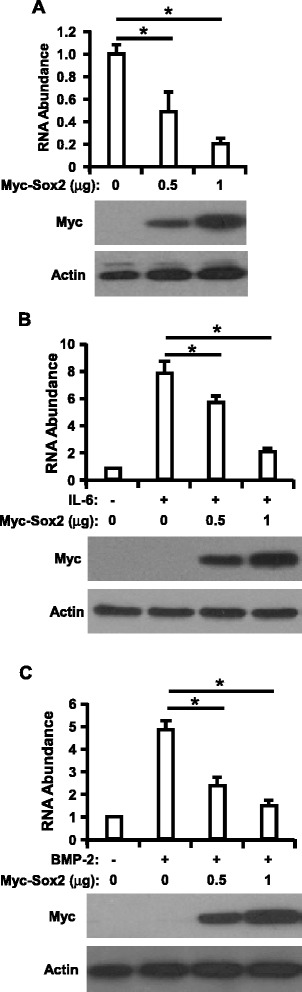
Overexpression of Sox2 can negatively regulate HAMP expression in Huh7 cells. **(A)** Huh7 cells were transfected with an increasing amount of plasmids expressing Myc-Sox2 (pcDNA3.1-Myc vector was used to balance the DNA to the same quantity in each group), and cell were harvested for RT-qPCR assay to determine HAMP expression and also cell lysates were analyzed with immunoblotting with the anti-Myc or anti-actin antibodies at 48 h post-transfection. **(B-C)** Huh7 cells were transfected with the plasmids expressing Myc-Sox2. Forty-eight hours later, cells were stimulated with IL-6 **(B)** or BMP-2 **(C)** for additional 6 hours. And cells were harvested for RNA extraction to determine HAMP expression and some cell lysates were immunoblotted with the anti-Myc antibody to analyze the Myc-Sox2 expression. The experiments were repeated for three times with similar results and data were shown as means ± SE. Statistical analyses were conducted using one-way ANOVA with Tukey’s multiple comparison test. Significant differences are indicated by *p < 0.05.

To examine the regulation of Sox2 on HAMP, we transfected Huh7 cells with increasing doses of plasmids expressing Myc-Sox2 and RNA was extracted from Huh7 cells and then analyzed HAMP expression level with RT-qPCR assay at 48 h posttransfection. We found that overexpression of Sox2 can dramatically reduce HAMP expression in Huh7 cells in a dose dependent manner (Figure 1A). To exclude our observation is cell specific, we also performed this assay in HepG2 cells, and we observed the similar phenomenon (Additional file 1: Figure S3).

It was reported that IL-6 and BMP-2 could up-regulates transcription of HAMP [5,17]. To further evaluate the inhibitory effect of Sox2 on HAMP expression, we transfected Sox2 into Huh7 cells prior to IL-6 or BMP-2 stimulation, and RT-qPCR assay was performed to determine HAMP expression, as presented in Figure 1B and C, treatment of IL-6 or BMP-2 led to an average of 7.8 or 4.7 folds increase of HAMP, respectively, but this was significantly impaired in Sox2 transfected Huh7 cells, which suggest that Sox2 can inhibit IL-6 or BMP-2 induced HAMP expression (Additional file 1: Figure S4).

### Knockdown of Sox2 increase HAMP expression in hepatoma-derived cells

**Figure 2:**
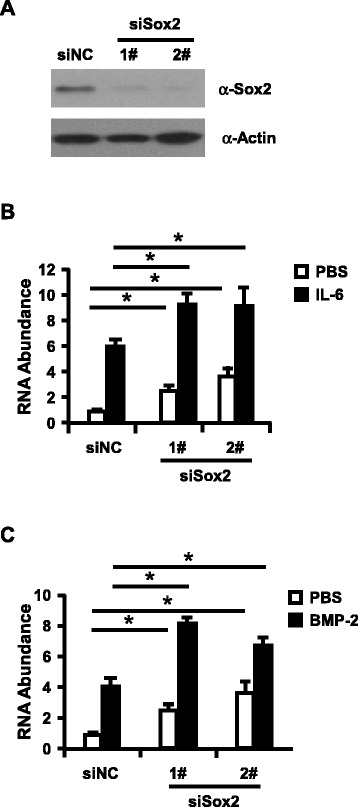
Knockdown of Sox2 can further increase HAMP expression in Huh7 cells. **(A)** Huh7 cells were transfected with Sox2-targeting siRNAs and control siRNA. Western blot analysis of partial Sox2 knockdown cells was performed to evaluate the Sox2 knockdown efficiency. **(B-C)** Huh7 cells transfected with Sox2 siRNAs were treated with IL-6 or BMP-2 were subjected for RNA isolation to determine HAMP expression with RT-qPCR assay. The experiments were repeated for three times with similar results and data were shown as means ± SE. Statistical analyses were conducted using one-way ANOVA with Tukey’s multiple comparison test. Significant differences are indicated by *p < 0.05.

To further test the role of Sox2 in regulation of HAMP expression, we knockdown Sox2 with siRNA prior to stimulation of Huh7 cells with IL-6 or BMP-2. Sox2 knockdown efficiency and HAMP expression were determined with Western Blotting assay (Figure 2A) or RT-qPCR assay. As illuminated in Figure 2B and C, IL-6 or BMP-2 can induce the expression level of HAMP about 6.0 or 4.1 folds respectively, and IL-6 or BMP-2 induced HAMP expression was further enhanced in Sox2 knockdown Huh7 cells (9.1 or 8.2 folds respectively), and knockdown of Sox2 can also slightly increased HAMP expression in the IL-6 and BMP-2 untreated cells. Taken together, all the data presented here support that Sox2 function as the negative regulator to control the HAMP expression. In the following assay, we ought to dissect the molecular mechanism of Sox2 regulation on HAMP expression.

### Sox2 regulate HAMP reporter activity

**Figure 3:**
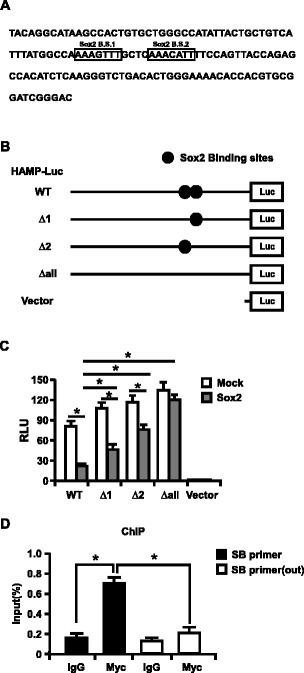
Sox2 binds with HAMP promoter to regulate its expression. **(A)** Two putative Sox2 binding sites were indicated with open boxes. **(B)** Schematic diagram of a series of HAMP promoter luciferase reporter constructs and putative Sox2 binding sites were marked. **(C)** Huh7 cells were cotransfected with various HAMP promoter luciferase reporter plasmids and the internal control plasmid pRL-TK, together with Myc-Sox2 or not. Forty-eight hours later, the cell lysates were subjected to dual luciferase assay, and the results were expressed as fold induction of luciferase activity relative to the empty vector without Myc-Sox2 expression. The error bar represented three replicates. **(D)** Huh7 cells transfected with Myc-Sox2 for 48 h were cross-linked, and sonicated to generate chromatin fragments. The sheared chromatins were immunoprecipitated with monoclonal antibodies against Myc or a mouse IgG isotype control followed by qPCR analysis using the primer set flanking the HAMP promoter. The data were expressed as the percentage of immunoprecipitated chromatin DNA versus the total input. The experiments were repeated for three times with similar results and data were shown as means ± SE. Statistical analyses were conducted using a one-way ANOVA with Tukey’s multiple comparison test to determine individual p-values **(C)** or an unpaired, two-tailed student t-test **(D)**. Significant differences are indicated by *p < 0.05.

To map the region in the HAMP promoter responsible for Sox2 exertion on HAMP transcriptional control, we analyzed the DNA sequences 1,000-bp upstream of HAMP start codon by TFsearch program (http://www.cbrc.jp) and TESS Tool (http://www.cbil.upenn.edu/cgi-bin/tess/tess). Two putative Sox2 binding sites were identified (Figure 3A). To confirm this bioinformatical result, we constructed a series of HAMP promoters (Figure 3B) upstream of a promoterless luciferase ORF in the pGL3-enhancer vector. The engineered HAMP-Luc plasmids were transfected into Huh7 cells together with Myc-Sox2 or not. The luciferase activities were measured to determine the HAMP promoter activity. As shown in Figure 3C, Sox2 can decrease HAMP promoter activity about 3.2 folds; in order to validate the roles of the potential Sox2 binding sites (highlighted in Figure 3A) in the HAMP promoter for Sox2 mediated regulation on HAMP expression, we individually or completely deleted the potential Sox2 binding sites, and then transfected them into Huh7 cells with or without Sox2. The data suggested that deletion of any Sox2 putative binding sites compromised Sox2 mediated decrease of HAMP promoter activity, and the Sox2 mediated inhibitory effect was completely impaired if both of the Sox2 putative binding sites were deleted, suggesting that both Sox2 binding sites are required for Sox2 exert inhibitory effect on HAMP expression.

### Sox2 can directly bind with HAMP promoter

To further analyze the binding of Sox2 with HAMP promoter, we performed ChIP assay. We transfected Myc-Sox2 into Huh7 cells and then harvested cells at 48 h post-transfection. Cells were processed and the cell lysates were immunoprecipitated with antibodies for Myc or isotype lgG, followed by qPCR using the primer sets (SB primers) specifically for the HAMP promoter sequence to quantify the amount of the HAMP bound DNA, and another primer sets (SB primer(out)) outside of the Sox2 putative binding sites were also used for the qPCR assay for control. As shown in Figure 3D, the amount of HAMP promoter precipitated by Myc increased for about 4-fold as compared to the isotype lgG control. These results revealed direct and specific binding of Sox2 to cis-regulatory sequences in the HAMP promoter region.

### Sox2 negatively regulate HAMP expression in primary hepatocytes

**Figure 4:**
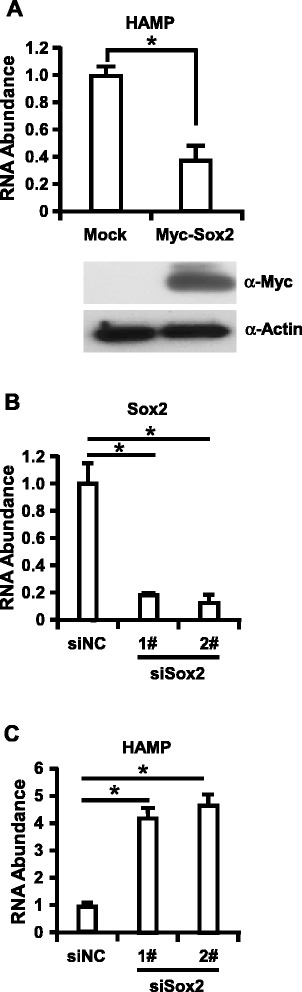
Sox2 negatively regulate HAMP2 expression in primary hepatocytes. Primary hepatocytes were cultured and lentivirus expressing Myc-Sox2 **(A)** or shRNA targeting Sox2 **(B and C)** infected the cells. Cells were harvested at 72 h later for Western blotting assay and RT-qPCR assay to determine Myc-Sox2 expression, HAMP expression and Sox2 knockdown efficiency. The experiments were repeated for three times with similar results and data were shown as means ± SE. Statistical analyses were conducted using a one-way ANOVA with Tukey’s multiple comparison test to determine individual p-values **(B-C)** or an unpaired, two-tailed student t-test **(A)**. Significant differences are indicated by *p < 0.05.

Finally, we sought to determine whether our observations could be verified in human primary hepatocytes. Firstly, we transduced the primary hepatocytes with lentivirus expressing Myc-Sox2. Cells were collected at 72 h postinfection and Immunoblotting assay was performed to analyze the expression of Myc-Sox and total intracellular RNA was extracted for RT-qPCR assay to detect the expression of HAMP. We found that lentivirus could efficiently transduce Myc-Sox2 expression in primary hepatocytes and overexpression of Myc-Sox2 could decrease HAMP expression with an average of 3 folds in primary hepatocytes (Figure 4A). Furthermore, we infected primary hepatocytes with shRNA targeting Sox2, cells were collected at 72 h postinfection, and RT-qPCR assay was performed to determine Sox2 knockdown efficiency and HAMP expression, as shown in Figure 4B and C, knockdown of Sox2 about at 80% efficiency increased HAMP expression level more than 4 folds in primary hepatocytes. Collectively, all the data demonstrated that Sox2 function as a negative regulator to control HAMP expression.

HAMP is a small, disulfide-rich peptide produced predominantly by the liver, and plays an important role in regulating systemic iron metabolism in mammals [18,19]. Under physiological conditions, the expression of HAMP is regulated by numerous factors [20]. The known positive regulators are the TfR2 (transferrin receptor 2) [21,22], hereditary HFE (haemochromatosis) protein [21], BMPs (bone morphogenetic proteins) [17,23,24] and HJV (haemojuvelin) [23-26]. One of negative regulators of liver hepcidin expression is Matriptase-2, a transmembrane serine protease, inhibits the expression of HAMP by interacting with membrane HJV and cleaving it into fragments [27-29]. In previous study, SMAD7 was identified as another negative regulator of HAMP gene expression [30]. SMAD7 is an inhibitory SMAD protein that mediates a negative feedback loop to both TGF-β (transforming growth factor β) and BMP signaling [31]. Sox2 is a member of the SOXB1 subfamily of high-mobility group box proteins and plays a key role in reprogramming somatic cells into pluripotent stem cells and neural stem cells in culture [16,32-35]. Our present study further demonstrates that Sox2 can negatively regulate the expression of HAMP, suggesting that Sox2 might be an important factor to control HAMP expression level to regulate systemic iron metabolism.

In the present study, we found that overexpression of Sox2 can decrease HAMP expression in hepatoma derived cells, and Sox2 can further downregulate IL-6 or BMP-2 induced HAMP expression in these cells (Additional file 1: Figure S4); in contrast, knockdown of Sox2 significantly increase HAMP expression in these cells. All the data support Sox2 as a negative regulator to control HAMP expression. We further constructed HAMP promoter driven reporter system and two putative Sox2 binding sites were identified in the promoter region. We found that the Sox2 binding sites are required for Sox2 to exert its inhibitory effect on the expression of HAMP, and ChIP assay also suggested that Sox2 can directly bind with the HAMP promoter region. Consistent with these results, we showed that the overexpression of Sox2 in human primary hepatocyte cells decreased HAMP expression, whereas knockdown Sox2 can enhance the expression of HAMP. Moreover, the negative regulation of Sox2 on HAMP should be verified in vivo to evaluate its role in the iron metabolism disorder, which is induced by high expression of HAMP. The molecular mechanism of Sox2 regulation of HAMP expression is unknown, and Sox2 has been found to upregulate Rex1 and Nanog with Oct3/4 [36,37]. In this study, we found that Sox2 function as the negative regulator for HAMP expression, which is different from the previous findings. In addition to Oct3/4, Sox2 was found to interact with other molecules, such as Pax6 [38] or NPM1 [39]. The different regulation of Sox2 on the target genes may be dependent on the components of the Sox2 associated complex in the nucleus.

Our discovery shed more light on the transcriptional regulation of HAMP by Sox2 and facilitates understanding the underlying mechanisms of maintaining body iron homeostasis. Interventions targeting this pathway could be useful for therapy of the resulting iron metabolism disorders.

## Conclusion

We found that Sox2 as a novel factor to bind with HAMP promoter to negatively regulate HAMP expression, which may be further implicated as a therapeutic option for the amelioration of HAMP-overexpression-related diseases, including iron deficiency anemia.

## Methods

### Cell culture

HEK293 cells, Huh7 cells, A549 cells, BxPC-3 cells, Hela cells, SV-HUC-1 cells, SW1353 cells and RWPE-2 cells, were grown in DMEM medium (Invitrogen, Carlsbad, CA, USA) containing L-glutamine, supplemented with 10% (vol/vol) heat-inactivated fetal calf serum, penicillin (100 units/ml), and streptomycin (100 mg/ml). The human hepatoma HepG2 cells were grown in RPMI 1640 media (Gibco) supplemented with the same components as mentioned above.

### Plasmid construction

To make the Myc-Sox2 expression construct, the cDNA encoding human Sox2 was cloned (nucleotide 438 nt to 1391 nt, GenBank Accession: NM_003106) into pcDNA3-Myc vector; and then the Myc-Sox2 was cloned into pLVX- IRES-Puro lentivirus vector to generate the pLVX-Myc-Sox2-IRES-Puro construct. To construct the HAMP promoter driven luciferase reporter plasmid (HAMP-Luc), a 1,000-bp sequence upstream of the translation start codon was amplified from genomic DNA of Huh7 cells using primers containing KpnI and HindIII sites. The PCR product was then cloned into pGL3-enhancer vector (Promega, Madison, WI, USA) [40]. All the Sox2 binding sites deleted mutants were generated by QuikChange® Site-Directed Mutagenesis Kit (Stratagene, La Jolla, CA, USA). All constructs were verified by DNA sequencing analysis. The primer information is available upon request.

### Transfection assay

Plasmid expressing Myc-Sox2, HAMP-Luc or siRNA targeting human Sox2 were transfected into target cells with Lipofectamine™ 2000 (Invitrogen) following manufacture’s instruction.

Lentiviruses expressing the Myc-Sox2 were obtained by co-transfecting pLVX-Myc-Sox2-IRES-Puro with the packaging and envelope vectors into HEK293T cells. Viral supernatants were harvested 48 h post transfection, filtered and used to infect primary hepatocytes.

### Immunoblot analysis

For western blot analysis, the cells were lysed in sample buffer (20 mM Tris–HCl, pH7.5, 150 mM NaCl, 1 mM EDTA, 1% NP40) containing Complete Protease Inhibitor Cocktail (Roche) and then incubated at 95°C for 10 min. Cell lysates were cleared by centrifugation and supernatants were separated by SDS–PAGE and analyzed by immunoblotting. The Luminata Forte Western HRP Substrate (Millipore) was used for the development of positive signals. The antibodies used in this study include Sox2(Abcam, #ab97959), Myc (Cell Signaling #9402) and beta-Actin (Cell Signaling #4970).

### RNA isolation and quantitative RT-PCR

RNA was isolated by TRIzol® Reagent (Life Technologies) in according to the manufacturer’s protocol. RNA pellets were resuspended in diethylpyrocarbonate (DEPC)-treated water and the RNA concentration was determined using the NanoDrop®ND-1000 spectrophotometer (Thermo Scientific). Complementary DNA (cDNA) was synthesized from 1 μg of RNA using random hexmers and TaqMan® Reverse Transcription Reagents (Applied Biosystems, Roche, NJ, USA) and amplified using primers specific for GAPDH (forward: 5′-GAA GGT GAA GGT CGG AGT C-3′; reverse: 5′-GAA GAT GGT GAT GGG ATT TC-3′ . Accession NO.: NM_002046; the length of the amplicon is 226 bp), and HAMP (forward: 5′- CGC TTG CCT CCT GCT CCT-3′; reverse: 5′- CTC GCC TCC TTC GCC TCT-3′. Accession NO.: NM_021175; the length of the amplicon is 152 bp) using SYBR® Green Real time PCR Master Mix (TOYOBO, Osaka, Japan). The specificity is determined by the melt curve analysis. For the data analysis, the Ct (threshold cycle) values for the gene of interested were normalized to those for GAPDH.

### Promoter assay

Ten thousand Huh7 cells were seeded into 48-well plates overnight prior to transfection with 20 ng/well of HAMP-Luc constructs and 10 ng/well of CMV promoter driven *Renilla* luciferase vector (pRL-CMV; Promega) together with Myc-Sox2 (0.5ug/well) or not. The cells were harvested 24 h later and cell lysates were assayed for luciferase activities using the Dual-Luciferase Reporter Assay System (Promega) following the manufacturer’s instructions.

### ChIP assay

Huh7 cells were transfected with Myc-Sox2 and cells were harvested at 48 h posttransfection. Cells were cross-linked, and sonicated to generate chromatin fragments according to a previously described protocol [41]. In particular, the cells were crosslinked with 1% formaldehyde, then lysed and sonicated to shear DNA. Sheared chromatins were then precleaned with protein A agarose. 20% of the sheared chromatins were kept as “input control”. The rest were incubated with antibodies against Myc (Sigma-Aldrich) or normal mouse IgG isotype control followed by additional incubation with protein A agarose. The bead-bound protein-DNA complexes were eluted and crosslinks were reversed. Precipitated DNA was subjected to qPCR using the HAMP promoter primers: 5′-CCA TAT TAC TGC TGT CAT TTA T-3′ and 5′-CAG TGT CAG ACC CTT GAG ATG-3′, and the control primers which were upstream of the Sox2 binding sites as the control: 5′-TTG CCC AGG CTA GTC TTG AA-3′ and 5′-AGC ACA GTG GCT TAT GCC TG-3′. Enrichment values were normalized with corresponding input control.

### Knockdown assay

For siRNA experiments, siRNA targeting human Sox2 (1#: 5′-GGT TGA CAC CGT TGG TAAT-3′; 2#:5′-TGC CGA GAA TCC ATG TATA-3′) was transfected into targeting cells and cells were harvested at indicated time point for further assay. Non-specific oligonucleotides (5-GATCTGATCGACACTGTAATG-3) with no significant homology to any mammalian gene sequence were used as non-silencing controls in all experiments.

Double-stranded oligonucleotides targeting human Sox2 were cloned into the pLSLG lentiviral vector. The sequences targeting human Sox2 are the same with siRNA sequence. HEK293T cells were transfected with the respective lentiviral vectors and packaging vectors to ensure proper viral packaging. Viral supernatants were harvested 48 h post transfection, filtered and used to infect primary hepatocyte cells to knockdown Sox2.

### Primary human hepatocytes culture, and lentivirus infection

Human hepatocytes, purchased from PromoCell, were maintained in Hepatocyte Growth Medium (PromoCell, HD, GRE). Cells were plated at a density of 1 × 10^5^ cells on 48-well plates coated with type 1 collagen. The produced lentiviruses for Myc-Sox2 transduction and Sox2 shRNA knockdown mentioned aboved were inoculated to primary human hepatocytes. Three days later the lentivirus-transduced cells were collected for RT-qPCR analysis of HAMP expression.

### Statistical analysis

Statistical analysis of the data was performed by using the GraphPad Prism software (GraphPad Software). Specific tests are noted in figure legends.

## Additional file

Additional file 1: Figure S1. HAMP expression profiles of cell types and tissues. Data represent HAMP gene profiles for the indicated human tissues and cell lines on the BioGPS Gene portal (biogps.org/) indicate that HAMP is highly and specifically expressed in liver. **Figure S2.** HAMP expressed in hepatocyte-derived cells and primary hepatocytes with high level. RT-qPCR assay was performed to determine HAMP mRNA expression. Data were presented as the relative ratio of HAMP to GAPDH. The experiments were repeated for three times and data were shown as means ± SE. **Figure S3.** Overexpression of Sox2 can negatively regulate HAMP expression in HepG2 cells. HepG2 cells were transfected with an increasing amount of plasmids expressing Myc-Sox2 (pcDNA3.1-Myc vector was used to balance the DNA to the same quantity in each group), and RT-qPCR assay was performed to determine HAMP expression and also cell lysates were analyzed with immunoblotting with the anti-Myc or anti-actin antibodies at 48 h post-transfection. The experiments were repeated for three times and data were shown as means ± SE. Statistical analyses were conducted using one-way ANOVA with Tukey’s multiple comparison test. Significant differences are indicated by *p < 0.05. **Figure S4.** Overexpression of Sox2 can negatively regulate HAMP expression in Huh7 cells. Huh7 cells were transfected with the plasmids expressing Myc-Sox2 (pcDNA3.1-Myc vector was used to balance the DNA to the same quantity in each group). Forty-eight hours later, cells were stimulated with IL-6 (A) or BMP-2 (B) for additional 6 hours. And RT-qPCR was performed to determine HAMP expression and some cell lysates were immunoblotted with the anti-Myc antibody to analyze the Myc-Sox2 expression. The experiments were repeated for three times and data were shown as means ± SE. Statistical analyses were conducted using one-way ANOVA with Tukey’s multiple comparison test. Significant differences are indicated by *p < 0.05.
