# Supplementary figures and images for: Sox2 function as a negative regulator to control HAMP expression
Source: Biol Res. 2015 May 6;48(1):23. doi: 10.1186/s40659-015-0013-z (PMC4440282; doi:10.1186/s40659-015-0013-z)

Supplemental Figure 1

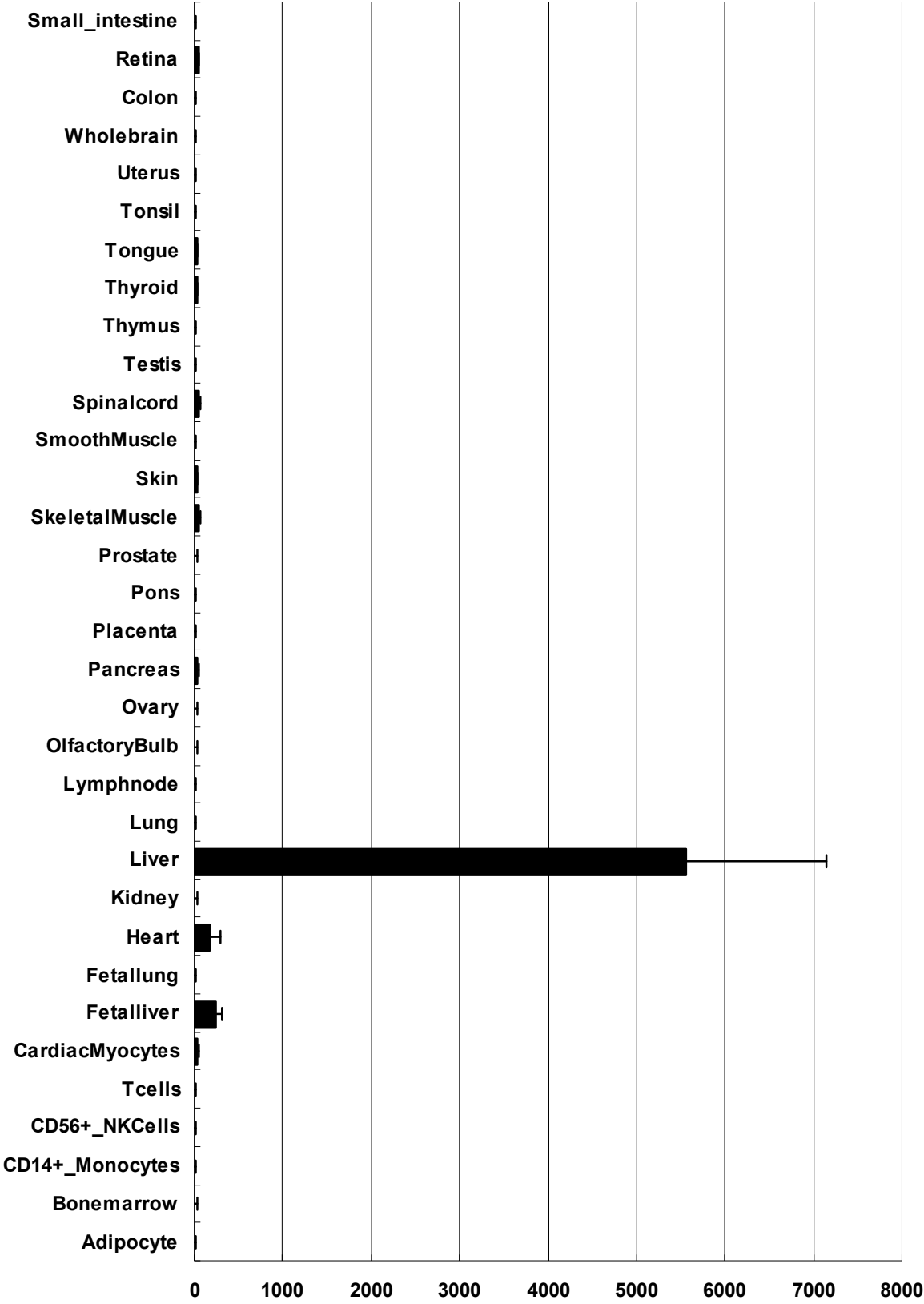

**Supplemental Figure 2**

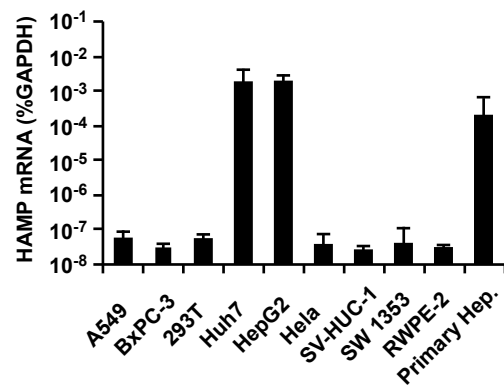

**Supplemental Figure 3**

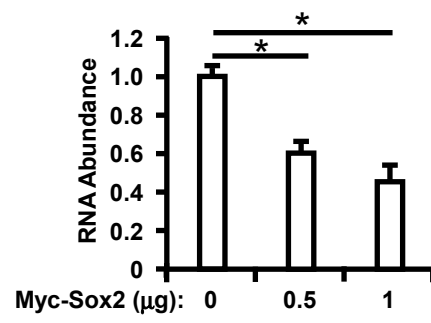

Supplemental Figure 4

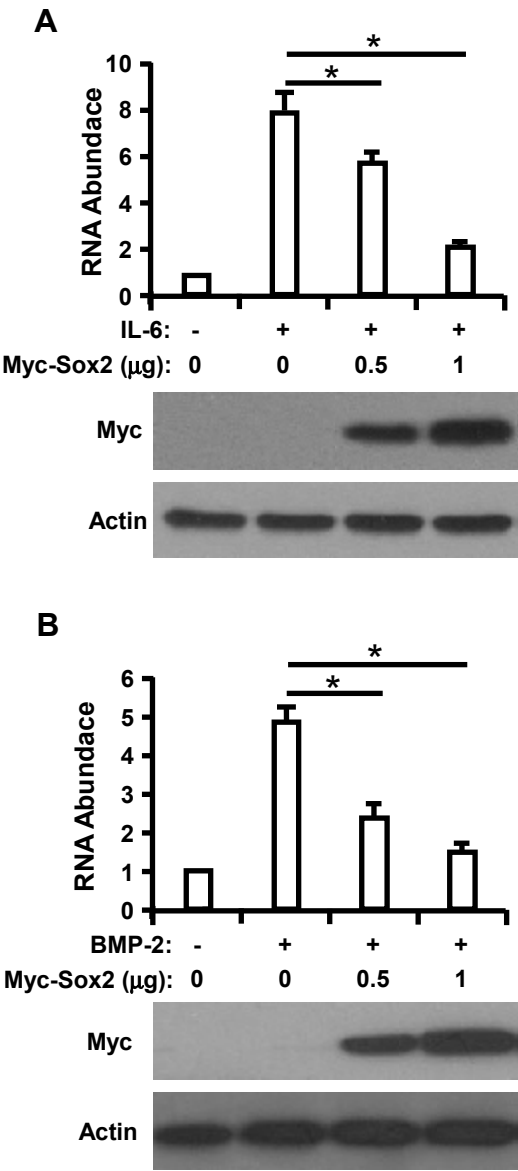

Supplement: Additional file 1: Figure S1. — HAMP expression profiles of cell types and tissues. Data represent HAMP gene profiles for the indicated human tissues and cell lines on the BioGPS Gene portal (biogps.org/) indicate that HAMP is highly and specifically expressed in liver. Figure S2. HAMP expressed in hepatocyte-derived cells and primary hepatocytes with high level. RT-qPCR assay was performed to determine HAMP mRNA expression. Data were presented as the relative ratio of HAMP to GAPDH. The experiments were repeated for three times and data were shown as means ± SE. Figure S3. Overexpression of Sox2 can negatively regulate HAMP expression in HepG2 cells. HepG2 cells were transfected with an increasing amount of plasmids expressing Myc-Sox2 (pcDNA3.1-Myc vector was used to balance the DNA to the same quantity in each group), and RT-qPCR assay was performed to determine HAMP expression and also cell lysates were analyzed with immunoblotting with the anti-Myc or anti-actin antibodies at 48 h post-transfection. The experiments were repeated for three times and data were shown as means ± SE. Statistical analyses were conducted using one-way ANOVA with Tukey’s multiple comparison test. Significant differences are indicated by *p < 0.05. Figure S4. Overexpression of Sox2 can negatively regulate HAMP expression in Huh7 cells. Huh7 cells were transfected with the plasmids expressing Myc-Sox2 (pcDNA3.1-Myc vector was used to balance the DNA to the same quantity in each group). Forty-eight hours later, cells were stimulated with IL-6 (A) or BMP-2 (B) for additional 6 hours. And RT-qPCR was performed to determine HAMP expression and some cell lysates were immunoblotted with the anti-Myc antibody to analyze the Myc-Sox2 expression. The experiments were repeated for three times and data were shown as means ± SE. Statistical analyses were conducted using one-way ANOVA with Tukey’s multiple comparison test. Significant differences are indicated by *p < 0.05. [file 40659_2015_13_MOESM1_ESM.pdf]
